# Research on the tracking effect of cooperative learning-based physical education teaching on college students' intrinsic motivation and psychological resilience

**DOI:** 10.3389/fpsyg.2026.1806429

**Published:** 2026-07-06

**Authors:** Hongyu Zheng, Shaoxuan Sun, Hui Sheng

**Affiliations:** 1 School of Physical Education, Shandong University, Weihai, Weihai, China; 2 Student Affairs Office, Harbin Institute of Technology Weihai, Weihai, China

**Keywords:** college students, cooperative learning, intrinsic motor motivation, physical education teaching, psychological resilience

## Abstract

For researching the long-time influence of cooperative learning sports teaching patterns on university students' inner drive for exercise and psychological toughness, we used cluster sampling to choose 160 participants which include 82 males and 78 females, then divided them into experiment group and control group, to carry out a 12-month teaching intervention research. Through questionnaire investigation, statistical analysis, and qualitative talks, we made systematic analysis on the dynamic change of students' inner exercise drive and psychological toughness under different teaching patterns. Thus, results showed that cooperative learning obviously lifted scores in all dimensions of inner exercise drive, with lasting long-time intervention effects. Therefore, it effectively developed psychological toughness, realizing all-round improvement in emotion control, pressure resistance, and goal persistence. A marked positive correlation was found between inner exercise drive and psychological toughness; hence, interactive quality, task design, and evaluation systems in cooperative learning had marked predictive functions for both two variables. This study uncovers the psychological cultivation mechanisms of cooperative learning, providing theory support and practice paths for reforming higher education institutions' physical education teaching.

## 1 Introduction

In this age when national sports strategy and comprehensive education reform carry out deep integration, university physical education is making a transition from traditional skill teaching work to the overall development of body and mind. College students confront multiple difficulties such as academic pressure and social adaptation, therefore physical education becomes a crucial tool for cultivating physical and mental health. Its value is not limited to the improvement of physical fitness; moreover, it also cultivates internal motivation for continuous exercise and psychological toughness to defeat difficulties. However, current university physical education still meets problems like inflexible teaching modes and insufficient interactive activities. Technical training with single focus cannot satisfy students' psychological development demands, thus leading to reduced exercise motivation and insufficient stress resistance in part of students.

Cooperative learning, being one interactive and collaborative teaching idea, uses group-based cooperation and task-led methods to build various interactive learning surroundings that fit college students' social development needs. Current research mostly centers on short-term motor skill improvements via cooperative learning, hence long-term studies about intrinsic motivation and psychological resilience are still not enough. Intrinsic motivation comes from people's interest and sense of belonging to sports activities, which acts as the core driving power for keeping on participating. Psychological resilience shows a person's ability to adapt and recover under pressure, and together they form the psychological base for college students' healthy development. This study combines internationally accepted theoretical frameworks—Self-Determination Theory, cooperative learning theory, and psychological resilience measurement theory—with real university physical education situations (Ryan and Deci, 2000; Johnson, 1970; Johnson and Johnson, 1975; Connor and Davidson, 2003). By using cooperative learning as an intervention tool and doing a 12-month long-time study, we analyze its long-term working ways to boost intrinsic motivation and psychological resilience. We combine embodied cognition and self-determination theories, thus we build a teaching logic structured as “context creation—interactive participation—psychological development.” Through multi-aspect data collection and deep analysis, this research finds out the special value of cooperative learning in stirring up intrinsic motivation and raising psychological resilience. The research results provide theoretical support and practical paths for the reform of physical education teaching in higher education schools, and aim to reach the educational goals of “cultivating wisdom through sports and nurturing hearts through sports.”

## 2 Literature review

### 2.1 Application research of cooperative learning in physical education teaching

Cooperative learning took its source in the 1970s American educational reform movement. Through group labor division, shared goal objects, and mutual outcome dependence, it nurtures learners' active participation behavior and collaborative development process (Johnson, 1970; Johnson and Johnson, 1975). In physical education domain, this method breaks away from the traditional "teacher leads, students are passive" teaching model pattern. Therefore, through collaborative completion of sports tasks, it cultivates students' communication capability, teamwork consciousness, and problem-solving skill abilities. Related studies have also discussed the development of cooperative learning and game-based teaching models in sports education (Benlian et al., 2021).

International researches show that cooperative learning can effectively raise the degree of participation in physical education classes. In the instruction of group projects, measures such as role assignment and tactical discussion may strengthen students' sports experiences and increase the effectiveness of skill acquisition. Domestic researches mainly focus on the construction of teaching models, for example, combining cooperative learning with sports like volleyball and basketball, thus proving its positive influence on technical ability and classroom atmosphere (Yang, 2015). However, current researches have obvious limitations: first, the intervention cycle is still short, typically 1-2 teaching cycles, with insufficient follow-up of long-term results. Second, research perspectives are relatively narrow, paying attention to skill improvement and short-term motivation while ignoring deeper psychological effects. Third, teaching implementation lacks specificity, being unable to design collaborative tasks sufficiently according to the characteristics of different sports and individual differences of students.

### 2.2 Influencing factors and cultivation pathways of intrinsic motivation in college students

Intrinsic motor motivation means one's psychological tendency to take part in physical activities, which is pushed by inner enjoyment, challenges, or the feeling of achievement linked with sports. Its core parts contain cognitive interest, achievement experience, and independent selection. Self-Determination Theory puts forward that satisfying autonomy, competence, and belongingness is very important for waking up intrinsic motor motivation; thus, the interactive nature of teaching environments and the rationality of task design have direct influence on the satisfaction of these psychological needs (Ryan and Deci, 2000). Hence, sports motivation research further makes sure that teaching environments with autonomous support have a long-lasting effect on raising intrinsic motor motivation (Vallerand, 2007; Reeve, 2015).

Current research has shown that students' sports participation, exercise atmosphere, project experience, and habitual exercise behavior are closely related to exercise motivation and exercise persistence (Yuanhui et al., 2022; Tao et al., 2022; Weina et al., 2011). Traditional instruction based on lecture usually reduces students' sense of self-control, but interactive teaching forms which stress learning through practice can effectively raise levels of inner movement motivation. Collaborative learning methods—via assigning group tasks and sharing final results—satisfy students' demand for making decisions by themselves. Therefore, by designing tasks with different levels, students can gradually beat difficulties together as a team, thus improving their sense of being capable. Team interactions and mutual help can strengthen their feeling of belonging, hence together promoting inner movement motivation. However, present studies have two shortcomings: first, they do not do enough research on specific paths through which collaborative learning elements such as task design, role distribution, evaluation mechanisms influence inner movement motivation; second, they lack long-term tracking analysis on the stability of inner movement motivation, thus preventing people from recognizing effects of long-lasting education interventions.

### 2.3 Research on the connotation of psychological resilience in college students and sports teaching intervention

Psychological resilience means a person's capability to keep positive adaptation and quick recovery when he or she confronts adversity, stress, or difficult situations. Its core aspects contain emotional control, stress bearing ability, and goal keeping. Sports activities, which have features of challenge and interactive movement, act as natural places to grow psychological resilience. Cooperative learning, via team support and working together to solve problems, strengthens the psychological development effects of physical education. Classic research in psychological resilience measurement and psychological capital theory has built internationally accepted models for evaluating and raising resilience (Connor and Davidson, 2003; Luthans et al., 2007). Therefore, studies on educational motivation further offer theoretical bases for teaching actions targeted at developing psychological qualities (Reeve, 2015).

Research shows that group-centered physical exercises can produce effective improvement on psychological resilience of college students. Existing studies have also suggested that physical exercise is associated with negative emotion regulation, psychological resilience, social support, mental health, and exercise self-efficacy among college students (Jingtao et al., 2022; Weijie, 2024; Yong et al., 2024; Xiangjuan et al., 2025; Kaitao, 2025). When college students make collaborative efforts to deal with difficult sports tasks, their stress resistance ability and emotional regulation capacity will get improvement. Applying cooperative learning method in physical education courses lets students obtain peer support and strategy exchange when they confront technical difficulties or competition pressure, thus they can gradually cultivate setback resilience and mental adjustment skills. However, current studies have obvious deficiencies: First, psychological resilience assessments mostly depend on single-item scales, which are short of multidimensional and dynamic measurement instruments. Therefore, second, the internal action mechanisms of cooperative learning's influence on psychological resilience are not analyzed enough, with unclear action paths related to interactive processes, task complexity, and support systems. Hence, third, long-term follow-up researches are very few, which blocks the verification of long-lasting effects brought by teaching interventions on psychological resilience development.

### 2.4 Research review

A full-scale analysis of existing researches shows that cooperative learning in physical education brings positive impacts to raising participation degrees and reinforcing skill acquisition, and meanwhile it may cultivate intrinsic exercise motivation as well as psychological resilience. Therefore, current researches face difficulties such as insufficient intervention length, limited research scopes, and inadequate mechanism analysis. Hence, the absence of long-term track studies on college students' intrinsic exercise motivation and psychological resilience causes difficulty to expose the lasting influence of cooperative learning teaching models.

This study carries out systematic checking of existing research gaps, selects cooperative learning as core intervention measure, and implements a 12-month long-term follow-up research to analyze its influence on college students' internal exercise motivation and psychological resilience. The innovative parts can be divided into three key items: First, lengthening intervention period and collecting data at multiple time nodes to inspect sustainability and dynamic changes of teaching effects. Second, optimizing research dimensions by splitting internal exercise motivation into three types—knowledge-pursuing interest, achievement feeling, and independent participation—and dividing psychological resilience into emotion regulation, pressure resistance, and goal adherence. Therefore, by integrating international long-term research results and meta-analysis conclusions, this study clarifies cooperative learning's action mechanisms and reinforces theoretical basis via evidence-based support. Third, enhancing empirical analysis through setting up control and experimental groups for comparative research, combining quantitative data with qualitative interview understandings to prove cooperative learning's educational value. Hence, these research outcomes provide targeted theoretical support and practical resolving schemes for physical education curriculum reform in higher education schools (Kaitao, 2025).

## 3 Study subjects and methods

### 3.1 Study subjects

By utilizing cluster sampling approach, we select 160 students who come from four Public Physical Education classes of the 2023 cohort in one university, among whom there are 82 male students and 78 female students with age ranging from 18 to 20. Through cluster randomization, the four classes are divided randomly into two groups each with 80 students, but individual randomization cannot be realized—this is one limitation which will be discussed in detail in the discussion section. The experimental group uses physical education teaching models based on cooperative learning, whereas traditional teaching methods are kept by the control group. Before the implementation of the intervention, pre-tests which assess intrinsic exercise motivation and psychological resilience have showed that there are no notable differences between the two groups in all dimensions, and this situation makes comparative analysis possible. Confounding variables such as students' previous sports experience, instructor's influence, or class arrangement are not controlled in this study; their potential effects will be examined in the discussion section henceforth.

### 3.2 Research tools

1. Intrinsic Motivation Scale for Physical Activity: It was developed on the foundation of core dimensions of exercise motivation from Vallerand and educational motivation measurement framework of Reeve, and then adapted so that it can match characteristics of collaborative learning (Vallerand, 2007; Reeve, 2015). It is composed of three dimensions—curiosity, achievement experience, and autonomous participation—with total 21 items. We use a 7-point Likert scale, thus higher scores can show stronger level of intrinsic motivation. First, three physical education experts conducted peer review for the revision process; next, a pilot test with *n* = 30 was carried out, hence item refinement was done according to feedback received. Cronbach's α coefficient of the scale is 0.876, and α coefficients for each dimension are in the range from 0.812 to 0.853. Confirmatory factor analysis found excellent fitting degree, therefore strong reliability and validity are confirmed.

2. Psychological Resilience Scale: It is revised according to the core framework of Connor-Davidson Resilience Scale (CD-RISC) and the Sports Psychological Resilience Questionnaire (Connor and Davidson, 2003). This scale has three dimensions, which are emotional regulation, stress resilience, and goal persistence, and it includes total 18 items measured by a 5-point Likert scale. Higher scores can show higher level of psychological resilience. After scale revision, expert validity testing and pilot testing have been carried out for optimizing item compatibility, hence the scale's Cronbach's α coefficient is 0.863, and the α coefficients of each dimension are in the range from 0.798 to 0.841. Confirmatory factor analysis has presented good fit indices, thus its reliability and validity can satisfy research requirements.

3. Semi-structured interview outline: We design interview questions according to themes like cooperative learning experiences, exercise motivation changes, and psychological resilience enhancement; therefore, our primary purposes are to supplement the insufficiency of quantitative data and to gain deeper comprehension about students' subjective perceptions and change processes hence.

### 3.3 Teaching intervention program

The teaching plan of the experimental group used a physical education model based on cooperative learning, with teaching content mainly centering on team sports like volleyball and basketball. Therefore, the intervention duration was 12 months, which included a total of 48 teaching sessions; thus, specialized teaching based on collaborative tasks was carried out every 2 weeks. Hence, the implementation process was divided into three phases.

The first phase is the foundational cooperation stage, it mainly centers on team establishment and rule learning. Through simple cooperation tasks like collective ball cushioning and group relay races, students' teamwork consciousness and communication abilities are cultivated. Every group is made up of 6-8 members arranged in heterogeneous configurations, and at the same time, it takes gender and athletic background differences into account.

The second stage centers on task deepening, it has layered cooperation activities like tactic discussion and team competition as its main features. Therefore, these practice activities make students raise their technical ability level and teamwork skill through carrying out complex tasks. Hence, a peer assessment system is put into practice for the purpose of strengthening self-management and feedback mechanism functions.

The third stage centers on application expansion, it has features of cross-group contests and sports-connected social practices for extending collaborative learning to outside classroom spaces. Therefore, this method develops students' self-organization abilities and lasting physical activity consciousness. Throughout the intervention period, thus, the implementation faithfulness of the collaborative learning model is strictly inspected via classroom observations, teacher records, and group feedback, therefore ensuring consistent teaching steps and matched task demands.

The teaching plan of control group used the traditional physical education model, mainly depending on teacher's explanations and demonstrations, with students carrying out uniform exercises. Therefore, the teaching content was the same as that of experimental group, but no specialized cooperative learning tasks were arranged. Hence, the control group only did routine group exercises without structured group interactions or collaborative tasks, and no informal group cooperation activities were carried out. Moreover, the evaluation method mainly was composed of technical proficiency tests.

### 3.4 Data collection and analysis methods

1. Data Collection: Before the experiment, three rounds of data collection activities were carried out, which were at the time point before experiment start (T0), at the mid-term phase of experiment (T1, 6 months), and at the experiment's ending time (T2, 12 months). Online questionnaires were used as the collection tool, with 160 copies issued in each round. The quantities of valid retrieved questionnaires were respectively 160, 156, and 152, so valid recovery rates respectively reached 100%, 97.5%, and 95%. Thus, the participant sample size decreased from 160 to 152. Drop-out analysis result confirmed that sample dropout was caused by random factors without systematic bias, hence this situation would not exert influence on the study's internal validity. At the experiment's conclusion, semi-structured interviews were implemented to 20 students coming from the experimental group, and each interview had a duration of 20–30 minutes. Besides, all interviews were recorded and then transcribed into textual form data.

2. Data Analysis Means: Data processing action was carried out by use of SPSS 27.0 and AMOS 24.0 software. The study's sample size was *n* = 160; this number was not fixed via statistical power analysis, and this method limitation will be talked about in the discussion part. Before data analysis, statistical presuppositions for parameter tests like t-tests and ANOVA were verified, which include normality examination, homogeneity of variance examination, and sphericity examination, with all data satisfying parameter testing's preconditions. Therefore, to consider clustering effects, cluster robust standard errors were used to make statistical correction for reducing independence assumption violations. Independent samples t-tests were applied to compare differences between experimental and control groups. Thus, repeated measures ANOVA was used to research experimental group performance changes across time points, and partial η^2^ effect sizes were reported for clarifying practical significance. Pearson correlation analysis was done to explore intrinsic motivation and psychological resilience's mutual relationship. Multiple regression analysis was utilized for revealing cooperative learning elements' predictive functions on intrinsic motivation and psychological resilience; prior to regression analysis, multicollinearity testing, standardized Beta coefficient calculations, and residual diagnostics were executed to guarantee sturdy results. Qualitative interview data were analyzed through coding techniques for extracting key themes, and representative interview excerpts were presented in structured thematic tables to make quantitative interpretation more convenient.

## 4 Research results and analysis

### 4.1 Analysis of differences in intrinsic exercise motivation between the experimental group and control group

1. Comparison of intrinsic exercise motivation scores between groups before the experiment (T0): Before the experiment, independent sample *t*-tests were carried out toward all dimensions of intrinsic exercise motivation for two groups. Results showed that between experimental group and control group, there are no notable differences in curiosity interest, achievement experience, autonomous participation, and total scores. Hence, it is indicated that initial intrinsic exercise motivation levels are consistent among groups. Therefore, a fair baseline for subsequent experimental interventions is established hereby (Table 1)

**Table 1:** Baseline comparison of intrinsic exercise motivation between the experimental and control groups at T0.

Dimension	Experimental group (*n* = 80)	Control group (*n* = 80)	*t* price	*p* price
Curiosity for knowledge	17.32 ± 3.45	17.18 ± 3.52	0.263	0.793
Achievement Experience	16.89 ± 3.21	16.75 ± 3.34	0.281	0.779
Independent participation	15.96 ± 3.18	15.82 ± 3.25	0.245	0.807
Total points	49.17 ± 8.63	48.75 ± 8.76	0.298	0.767

2. Comparison of intrinsic motivation scores between groups at mid-experiment stage (T1): After 6 months of instructional intervention, independent samples *t*-test results demonstrated that the experimental group scored significantly higher than the control group in knowledge-seeking interest, achievement experience, autonomous participation, and total scores. Notably, the dimensions of knowledge-seeking interest and autonomous participation showed statistically significant differences, indicating that the cooperative learning instructional model has demonstrated immediate efficacy in stimulating intrinsic motivation (Table 2).

**Table 2:** Comparison of intrinsic exercise motivation between the experimental and control groups at T1.

Dimension	Experimental group (*n* = 78)	Control group (*n* = 78)	*t* price	*p* price
Curiosity for knowledge	19.87 ± 3.12	17.56 ± 3.48	4.528	0.000
Achievement Experience	18.95 ± 3.05	17.23 ± 3.26	3.364	0.001
Independent participation	18.63 ± 2.98	16.15 ± 3.17	5.012	0.000
Total points	57.45 ±7.86	50.94 ± 8.23	4.876	0.000

3. Comparison of intrinsic exercise motivation scores between groups at the end of the experiment (T2): After 12 months of instructional intervention, independent samples t-test results demonstrated that the experimental group achieved significantly higher scores in all dimensions and total score of intrinsic exercise motivation compared to the control group. The differences further widened compared to the mid-experiment period, indicating that cooperative learning has sustained effects on stimulating intrinsic exercise motivation, with more pronounced long-term intervention outcomes (Table 3).

**Table 3:** Comparison of intrinsic exercise motivation between the experimental and control groups at T2.

Dimension	Experimental group (*n* = 76)	Control group (*n* = 76)	*t* price	*p* price
Curiosity for knowledge	22.45 ± 2.87	17.89 ± 3.35	8.236	0.000
Achievement Experience	21.32 ± 2.76	17.56 ± 3.18	7.154	0.000
Independent participation	20.87 ± 2.65	16.43 ± 3.09	8.962	0.000
Total points	64.64 ± 7.23	51.88 ± 8.05	8.573	0.000

4. Longitudinal Analysis of Intrinsic Motivation Changes in the Experimental Group: A repeated-measures ANOVA was conducted on intrinsic motivation scores at three time points. Results demonstrated that as intervention duration extended, the experimental group exhibited statistically significant improvements in curiosity interest, achievement experience, autonomous participation, and overall scores. Notably, the most substantial enhancement occurred in the autonomous participation dimension, indicating that collaborative learning has a pronounced long-term impact on cultivating intrinsic motivation (Table 4).

**Table 4:** Longitudinal changes in intrinsic exercise motivation in the experimental group across T0, T1, and T2.

Dimension	Before the experiment (T0)	Mid-experiment phase (T1)	At the end of the experiment (T2)	*F* price	*p* price	*post hoc* analysis (T0-T1)	*post hoc* analysis (T1-T2)
Curiosity for knowledge	17.32 ± 3.45	19.87 ± 3.12	22.45 ± 2.87	68.452	0.000	0.000	0.000
Achievement Experience	16.89 ± 3.21	18.95 ± 3.05	21.32 ± 2.76	57.328	0.000	0.001	0.000
Independent participation	15.96 ± 3.18	18.63 ± 2.98	20.87 ± 2.65	75.683	0.000	0.000	0.000
Total points	49.17 ± 8.63	57.45 ± 7.86	64.64 ± 7.23	72.895	0.000	0.000	0.000

### 4.2 Analysis of differences in psychological resilience between the experimental group and control group

1. Comparison of psychological resilience scores between the two groups before the experiment (T0): The independent samples *t*-test results prior to the experiment showed no significant differences in psychological resilience across all dimensions or total scores between the experimental group and the control group, indicating that the initial psychological resilience levels of the two groups were consistent, meeting the requirements of the experimental design (Table 5).

**Table 5:** Baseline comparison of psychological resilience between the experimental and control groups at T0.

Dimension	Experimental group (*n* = 80)	Control group (*n* = 80)	*t* price	*p* price
Emotional control	13.25 ± 2.76	13.12 ± 2.83	0.275	0.784
Stress tolerance	12.89 ± 2.63	12.75 ± 2.71	0.293	0.770
Goal persistence	14.12 ± 2.58	14.03 ± 2.65	0.218	0.828
Total points	40.26 ± 7.23	39.90 ±7.36	0.286	0.775

2. Comparison of psychological resilience scores between groups at mid-experiment stage (T1): After 6 months of instructional intervention, independent samples *t*-test results showed that the experimental group achieved significantly higher scores in emotion regulation and stress resistance dimensions compared to the control group, while no significant difference was observed in goal persistence dimension scores. These findings indicate that cooperative learning has exerted positive short-term effects on emotional regulation and stress coping dimensions of psychological resilience (Table 6).

**Table 6:** Comparison of psychological resilience between the experimental and control groups at T1.

Dimension	Experimental group (*n* = 78)	Control group (*n* = 78)	t price	p price
Emotional control	15.36 ± 2.54	13.52 ± 2.78	4.125	0.000
Stress tolerance	14.78 ± 2.46	13.15 ± 2.63	3.876	0.000
Goal persistence	14.89 ± 2.43	14.32 ± 2.57	1.428	0.156
Total points	45.03 ± 6.87	40.99 ± 7.12	3.689	0.000

3. Comparison of psychological resilience scores between the two groups at the end of the experiment (T2): After 12 months of teaching intervention, independent sample *t*-test results showed that the experimental group scored significantly higher than the control group in all dimensions and total score of psychological resilience, with a statistically significant difference also observed in the goal persistence dimension. These findings indicate that long-term cooperative learning interventions can comprehensively enhance students' psychological resilience levels (Table 7).

**Table 7:** Comparison of psychological resilience between the experimental and control groups at T2.

Dimension	Experimental group (*n* = 76)	Control group (*n* = 76)	*t* price	*p* price
Emotional control	17.54 ± 2.32	13.87 ± 2.65	8.012	0.000
Stress tolerance	16.89 ± 2.25	13.45 ± 2.58	7.653	0.000
Goal persistence	16.98 ± 2.17	14.56 ± 2.43	6.789	0.000
Total points	51.41 ± 6.23	41.88 ± 6.95	8.234	0.000

4. Longitudinal Analysis of Psychological Resilience Changes in the Experimental Group: Repeated-measurement ANOVA results demonstrated that all dimensions and total scores of psychological resilience in the experimental group exhibited statistically significant upward trends across three time points. The emotion regulation dimension showed the most substantial improvement, while the goal persistence dimension demonstrated accelerated growth rates from the mid-term to the final stage of the experiment. These findings indicate that cooperative learning facilitates a progressive enhancement of psychological resilience, with long-term interventions achieving comprehensive improvement in resilience levels (Table 8).

**Table 8:** Longitudinal changes in psychological resilience in the experimental group across T0, T1, and T2.

Dimension	Before the experiment (T0)	Mid-experiment phase (T1)	At the end of the experiment (T2)	*F* price	*p* price	*post hoc* analysis (T0-T1)	*post hoc* analysis (T1-T2)
Emotional control	13.25 ± 2.76	15.36 ± 2.54	17.54 ± 2.32	65.432	0.000	0.000	0.000
Stress tolerance	12.89 ± 2.63	14.78 ± 2.46	16.89 ± 2.25	58.765	0.000	0.000	0.000
Goal persistence	14.12 ± 2.58	14.89 ± 2.43	16.98 ± 2.17	42.348	0.000	0.156	0.000
Total points	40.26 ± 7.23	45.03 ± 6.87	51.41 ± 6.23	63.567	0.000	0.000	0.000

### 4.3 Correlation analysis between intrinsic motivation and psychological resilience

To investigate the relationship between intrinsic exercise motivation and psychological resilience, Pearson correlation analysis was conducted on two indicators in the experimental group at the end of the study. The results demonstrated significant positive correlations between all dimensions of intrinsic exercise motivation and psychological resilience. Notably, achievement experience showed the strongest correlation with stress resistance capacity, followed by autonomous participation and emotional regulation. These findings indicate that enhanced intrinsic exercise motivation significantly promotes psychological resilience development, establishing a mutually reinforcing positive relationship between the two factors (Table 9).

**Table 9:** Correlation between intrinsic exercise motivation and psychological resilience at T2.

Dimension	Emotional control	Stress tolerance	Goal persistence	Total score of psychological resilience
Curiosity for knowledge	0.683^**^	0.652^**^	0.598^**^	0.675^**^
Achievement Experience	0.725^**^	0.786^**^	0.693^**^	0.768^**^
Independent participation	0.754^**^	0.712^**^	0.657^**^	0.743^**^
Total score of intrinsic motivational drive	0.762^**^	0.778^**^	0.689^**^	0.815^**^

^**^p < 0.01.

### 4.4 Predictive role of collaborative learning elements on intrinsic motivation and psychological resilience

Using the three core elements of cooperative learning (task design, interaction quality, and evaluation mechanisms) as predictor variables, and the total scores of intrinsic exercise motivation and psychological resilience as dependent variables respectively, a multiple regression analysis was conducted. The results demonstrated that task design, interaction quality, and evaluation mechanisms could significantly predict scores in intrinsic exercise motivation and psychological resilience. Specifically, the regression results for intrinsic exercise motivation are shown in Table 10, while the regression results for psychological resilience are shown in Table 11.

**Table 10:** Multiple regression analysis of cooperative learning elements predicting intrinsic exercise motivation.

Predictive variable	Regression coefficient	Standard error	*t* price	*p* price	*R* ^2^	Adjust *R*^2^	*F* price
Task Design	0.287	0.065	4.415	0.000	0.689	0.678	62.345
Interaction quality	0.356	0.072	4.944	0.000			
Evaluation Mechanism	0.213	0.058	3.672	0.000			

**Table 11:** Multiple regression analysis of cooperative learning elements predicting psychological resilience.

Predictive variable	Regression coefficient	Standard error	*t* price	*p* price	*R* ^2^	Adjust *R*^2^	*F* price
Task Design	0.287	0.065	4.415	0.000	0.689	0.678	62.345
Interaction quality	0.356	0.072	4.944	0.000			
Evaluation Mechanism	0.213	0.058	3.672	0.000			

### 4.5 Analysis of qualitative interview results

We carried out coding analysis upon interview data coming from 20 experimental group students, and we identified three central themes. The thematic coding results are presented in Table 12. First of all, collaborative learning has raised the pleasure degree of sports experiences. Via group interactions and teamwork, during physical activities, students obtained more positive feedback and emotional support, which can effectively stir up their participation interest. Second, the challenge-overcoming process in team collaboration has helped students to develop gradually mental resilience, mutual support skills, and also promoted their stress management capabilities. Third, self-directed task distribution and shared results have lifted students' sense of autonomy and achievement, thus nurturing their sustained exercise motivation. Therefore, quantitative data has corroborated the interview findings, and it also demonstrates that collaborative learning brings positive influence to intrinsic exercise motivation and psychological resilience.

**Table 12:** Thematic coding results of semi-structured interviews in the experimental group.

Predictive variable	Regression coefficient	Standard error	*t* price	*p* price	*R* ^2^	Adjust *R*^2^	*F* price
Task Design	0.324	0.071	4.563	0.000	0.657	0.645	58.762
Interaction quality	0.298	0.068	4.382	0.000			
Evaluation Mechanism	0.187	0.061	3.065	0.003			

## 5 Discussion

### 5.1 Mechanism of cooperative learning on intrinsic motivation in college students

The research results prove that cooperative learning obviously raises college students' intrinsic exercise motivation, with long-term intervention showing especially strong effects—a conclusion that is in line with existing studies. Through analysis by self-determination theory, there are three mechanisms for cooperative learning to stimulate intrinsic motivation: it matches students' autonomy sense, it helps group task distribution and activity choice, and it frees learners from passive learning modes in traditional teaching methods. This method lets learning progress be self-directed, strengthens exercise control awareness, and at the same time improves competence. Gradual task design makes students together overcome technical difficulties, where group support and feedback let timely progress tracking be carried out, thus reinforcing positive views on sports ability and belonging feeling. Team interactions with mutual encouragement, shared successes, and collective recognition build emotional links with physical activities. Multidimensional analysis finds that autonomous participation dimension has the most obvious improvement, which shows cooperative learning has best effect in developing voluntary engagement. This result comes from increased decision-making autonomy—such as designing group strategies and role assignments—that changes learning situation from “being forced to learn” to “voluntary learning.” Score changes from middle to late experiment stages show intrinsic motivation has continuous growth momentum even though initial growth slows down, proving cooperative learning's lasting influence on intrinsic exercise motivation cultivation. This lasting effect is from a virtuous cycle between exercise interest and achievement experiences: positive experiences from autonomous participation can strengthen exercise interest, and continuous participation driven by interest leads to more achievement experiences, therefore consolidating intrinsic exercise motivation.

### 5.2 Pathways of cooperative learning in shaping psychological resilience among college students

Cooperative learning shows big effects on lifting college students' psychological resilience, getting step-by-step development from partial to all-around aspects. The most easy-to-see improvements happen in emotional regulation and stress resistance abilities, because team exchanges inside collaborative learning settings offer students places to express and adjust emotions. When meeting technical difficulties or competition pressures, peer support and strategy sharing can effectively reduce anxiety and help students build rational ways to deal with setbacks. Although the goal persistence part had no obvious improvement in the experiment's mid-term period, it showed very clear differences by the final stage. Therefore, this shows that training psychological resilience in goal persistence needs longer training time, hence it has connection with continuous task involvement and result feedback. The complex tasks and team goals inside long-term cooperative learning let students slowly master obstacle overcoming and persistence through non-stop efforts.

The core route to raise psychological resilience via cooperative learning rests on the double functions of “challenge-support”: First, graded tasks and team contests in collaborative study build middle-level difficulties; when encountering barriers, these push students to non-stop break their psychological comfort zones, therefore improving stress resilience. Second, teammates' support, encouragement and help offer psychological safety, decreasing fear and retreat behavior in challenge process, hence urging students to take risks and keep going. This “challenge and support act together” environment fully fits the central demands for growing psychological resilience, and step by step forms students' emotional adjustment skills, stress handling abilities, and goal stick-to capability.

### 5.3 Interaction between intrinsic motor motivation and psychological resilience

Correlation analysis has discovered a notably positive connection between inner sports drive and psychological toughness, thus showing a relationship where each side strengthens the other (Fangwu and Weiqiang, 2024; Jingdong, 2024; Mingke, 2024). Therefore, increased inner sports drive offers dynamic backing for the development of psychological toughness. When students join sports activities actively because of interest and achievement experiences, they will become more ready to face challenges in physical exercises, and thus they can improve their psychological toughness step by step through non-stop practice. Conversely, raised psychological toughness can further strengthen inner sports drive. Students who have strong ability of emotional control and stress resistance can deal better with setbacks related to sports, reduce the behaviors of giving up caused by difficulties, and thus keep their continuous interest in taking part in sports.

Achievement experiences show the most powerful correlation with stress resilience; this indicates that the accomplishment feeling students obtain via physical activities can raise their confidence and pressure-coping abilities in a significant way. Therefore, better stress resilience lets students show stronger toughness when they face technical difficulties, hence producing more achievement experiences. Thus, this mutually strengthening relation lets collaborative learning indirectly push forward psychological resilience development by activating inner motivation. At the same time, the cultivation of psychological resilience can further strengthen inner motivation, so building a positive cycle that combines physical and mental development together.

### 5.4 Priority levels of elements in collaborative learning

Multiple regression analysis discovers that interaction quality shows the most powerful predictive ability toward intrinsic exercise motivation, meanwhile, task design has the largest influence on psychological resilience. Therefore, these findings emphasize two key aspects in collaborative learning: interaction quality acts as the core driving force of intrinsic motivation, hence task design plays a crucial part in forming psychological resilience. The core of effective interaction is situated in real communication, meaningful support, and constructive feedback. High-quality interactions let students receive respect and recognition, thus cultivating emotional links and self-led engagement. Task design mainly includes difficulty advancement and collaborative requirements—moderate challenges stir up the wish for challenge, whereas strong collaborative needs push team unity and strategy sharing, therefore finally improving psychological resilience.

The assessment mechanism shows very strong predictive ability when it judges inner drive and psychological toughness, hence scientific assessment methods can raise the effect of cooperative learning. Methods like peer assessment and formative evaluation in collaborative learning have departed from the traditional single-method assessment system in physical education. These methods not only pay attention to skill progress but also stress cooperation ability and participation attitude, thus letting students obtain all-round recognition and confirmation. Therefore, this method can effectively stimulate inner drive and build up psychological toughness.

## 6 Conclusion

Through one 12-month longitudinal investigation, this research carries out systematic examination on the influence that cooperative learning in physical education exerts upon college students' intrinsic exercise motivation and psychological resilience. Therefore, key results are as follows: Cooperative learning can raise intrinsic exercise motivation in a significant way, hence interventions of long duration can show more steady effects. It can effectively promote three dimensions, which are curiosity for knowledge obtaining, achievement experience, and autonomous participation. Thus, this method also shows gradually increasing effectiveness in psychological resilience cultivation, and it can realize all-around improvements in emotional adjustment, stress resistance, and goal perseverance. It is noticeable that intrinsic exercise motivation and psychological resilience have positive correlation and mutually strengthening relationships. Interactive quality, task design, and evaluation mechanisms in cooperative learning models can significantly predict the development of both two dimensions. Among these factors, interactive quality produces the strongest effect on intrinsic exercise motivation, while task design is the most critical one for psychological resilience development.

The theoretical contribution of this study is to make clear the long-time mechanisms through which cooperative learning produces effects on college students' intrinsic exercise motivation and psychological resilience. Existing studies in physical education pedagogy and psychological development are enriched by this research; therefore, new empirical evidence is also provided for self-determination theory's application in sports instruction. Practical implications point out that higher education institutions ought to actively use cooperative learning models by optimizing task design, lifting interaction quality, and improving evaluation mechanisms to build up a “self-directed-cooperative-development” teaching environment. Hence, emphasis shall be laid on long-term progression and tiered learning methods, increasing task complexity and collaborative demands step by step to promote continuous advances in intrinsic exercise motivation and psychological resilience. Thus, special attention must be paid to individual student differences, and balanced consideration of gender and athletic background gaps must be ensured during group formation and task allocation processes, so as to guarantee positive learning experiences for all cooperative learning participants.

This research also owns definite limitations: the sample was solely selected from one single university, thus leading to restricted representativeness and failing to consider differences among various sports disciplines. Therefore, the effect of cooperative learning may change according to project-specific features. Additionally, the comparatively small sample size in qualitative research could not sufficiently capture subjective feelings of different groups. Hence, future researches may expand the sample range by including students from multiple universities and various sports disciplines. This can realize deep analysis of implementation ways and effectiveness differences of cooperative learning in different sports, at the same time integrate more qualitative research methods to fully show the micro-level processes through which cooperative learning affects intrinsic exercise motivation and psychological resilience.

## Data Availability

The original contributions presented in the study are included in the article/supplementary material, further inquiries can be directed to the corresponding author.
